# Rupatadine as an Add-On Therapy for Dengue Hemorrhagic Fever: A Case Report

**DOI:** 10.7759/cureus.106343

**Published:** 2026-04-02

**Authors:** Mauricio E Flores

**Affiliations:** 1 Pediatrics Allergy and Immunology, Clinica de Asma y Alergias, Santa Elena, SLV; 2 Pediatrics Allergy and Immunology, Hospital Nacional San Rafael, Santa Tecla, SLV

**Keywords:** dengue hemorrhagic fever (dhf), platelet-activating factor, rupatadine, supportive therapy, thrombocytopenia

## Abstract

Dengue hemorrhagic fever (DHF) is a severe form of dengue fever. Plasma leakage is the main pathophysiological hallmark that distinguishes DHF from mild dengue infection, and can result in hypovolemic shock and fatal outcome. No specific drugs and vaccines are available to treat dengue disease, and supportive therapy based on fluid replacement therapy and symptomatic medication (e.g., antipyretic/analgesics) should be promptly instituted. Platelet-activating factor (PAF) is associated with an increase in vascular permeability and has been found to be elevated in patients with DHF, supporting the use of strong PAF antagonists, such as rupatadine, in the management of severe dengue. We here report the case of a 17-year-old female patient presenting with fever, abdominal pain, headache, nausea, transvaginal bleeding, and thrombocytopenia (platelet count 45,000 cells/mm^3^), who was diagnosed with DHF by positive dengue IgM and IgG antibodies on the fifth day of illness. The patient was hospitalized, and oral rupatadine 40 mg/day was added to fluid-based supportive therapy. After two days of inpatient treatment, symptoms rapidly improved, and the platelet count increased to 110,000 cells/mm^3^. Nasal testing for SARS-CoV-2 infection performed on two occasions was negative. In summary, rupatadine as an add-on therapy to conventional therapy could reduce the risk of complications in DHF. This off-label use of rupatadine needs to be explored in larger studies.

## Introduction

Dengue is an arthropod-borne virus (arbovirus) caused by four genetically distinct viral serotypes, dengue virus (DENV) 1-4, and transmitted primarily by *Aedes aegypti* and *Aedes albopictus* mosquitoes. Dengue virus has been estimated to cause up to 390 million infections and 96 million symptomatic cases annually [1]. Also, due to the expansion of *Aedes *mosquitoes’ population related to climate change and globalization, dengue importation from tropical and subtropical zones to temperate areas constitutes an alarming public health threat [1]. The age-standardized incidence rate showed an overall increase of 47.3% from 1992 to 2021, with sub-Saharan Africa, Asia, Latin America, and the Caribbean as high-risk regions worldwide [2]. The Americas have experienced atypically intense dengue activity since 2019, with shifts in epidemic seasons and a greater proportion of severe cases and mortality in younger age groups [3].

Most patients with dengue have a self-limiting, febrile illness with a low mortality (<1%) when detected early and provided appropriate medical care. Dengue hemorrhagic fever (DHF) and dengue shock syndrome (DSS) are severe manifestations of the disease, associated with a mortality rate of around 2%-5% after receiving treatment but increasing to 20% if left untreated [4]. Vascular leakage is a hallmark of DHF and is thought to occur due to endothelial dysfunction resulting in increased vascular permeability. Patients with severe plasma leakage may have pleural effusions, ascites, hypoproteinemia, or hemoconcentration that can progress to life-threatening hypovolemic shock and increased risk of multi-organ failure [5]. Virus virulence, preexisting dengue antibodies, immune dysregulation, lipid change, and host genetic susceptibility are factors reported to be correlated with the development of DHF, but the underlying mechanisms that trigger DHF remain unclear [6].

There are no definitive curative medications for dengue. Careful monitoring and supportive management with fluid resuscitation during the critical phase is the cornerstone of treatment [7,8]. Platelet-activating factor (PAF), a potent phospholipid mediator mainly synthesized by the endothelial cells that disrupts endothelial tight junctions and amplifies cytokine production, has been shown to be elevated in severe dengue infections [9]. Therefore, drugs that can prevent or reduce vascular leakage, such as PAF antagonists, would be a suitable option to reduce symptoms in severe dengue and associated complications.

This case highlights the efficacy of rupatadine, a dual-acting drug with strong H1 receptor antagonism and potent PAF inhibition, in a young patient with DHF.

## Case presentation

A 17-year-old female patient from El Salvador consulted with a three-day history of moderate fever (38°C or above), persistent headache, abdominal pain, and nausea. The patient was evaluated using telehealth (July 2020) during the COVID-19 pandemic. She reported a personal history of polycystic ovary syndrome and obesity, as well as a family history of hypercholesterolemia and allergic rhinitis. Initially, the patient was treated with paracetamol as symptomatic antipyretic and analgesic medication together with probiotics. It was recommended to perform a rapid nasal COVID-19 test. On the fourth day of illness, the patient reported that the COVID-19 test result was negative, but the fever persisted. She also reported an episode of transvaginal bleeding unrelated to her normal menstrual cycle. A series of diagnostic tests was requested to evaluate the presence of other diseases. On the fifth day of illness, the fever disappeared (with the last peak at six hours before consultation), although abdominal pain was persistent. Results of laboratory tests provided via telemedicine showed a normal blood cell count, except for a low platelet count of 45,000 cells/mm^3^, a slight increase of aspartate aminotransferase (AST; 55 IU/L, normal range 0-32 IU/L), and positive dengue IgM and IgG antibodies (Table 1).

**Table 1:** Results of laboratory tests on the fifth day of illness

Laboratory test	Results	Reference range
Complete blood cell count		
Hemoglobin	14.9 g/dL	12-17 g/dL
Hematocrit	42.5%	37%-51%
Total leukocyte count	6,110 cells/mm^3^	4,500-10,000 cells/mm^3^
Polymorphonuclear neutrophils	36.5%	37%-70%
Lymphocytes	51.2%	20%-50%
Monocytes	10.3%	0%-8%
Eosinophils	0.3%	0%-6%
Platelet count	45,000 cells/mm^3^	150,000-450,000 cells/mm^3^
Biochemistry profile		
Aspartate aminotransferase	55 IU/L	0-32 IU/L
Alanine aminotransferase	30.4 IU/L	0-41 IU/L
Serum creatinine	0.73 mg/dL	0.5-0.9 mg/dL
Serology	Positive IgM and IgG dengue antibodies	Negative/positive/equivocal
SARS-CoV-2 rapid nasal antigen test	Negative	Negative/positive/equivocal

Based on these findings, including thrombocytopenia and clinical ascites as evidence of plasma leakage, a diagnosis of DHF was established, consistent with World Health Organization (WHO) criteria. The patient was admitted to the hospital for monitoring of vital signs, administration of fluid replacement therapy in accordance with dengue management guidelines [8], analgesics, esomeprazole, and oral rupatadine 40 mg daily as an add-on therapy, previously discussed with her mother. The patient underwent routine clinical and laboratory monitoring during hospitalization. Abdominal ultrasound or other imaging studies were not performed due to healthcare restrictions during the COVID-19 pandemic. No specific cardiac monitoring was deemed necessary, as there were no clinical indications for such evaluation.

**Figure 1:**
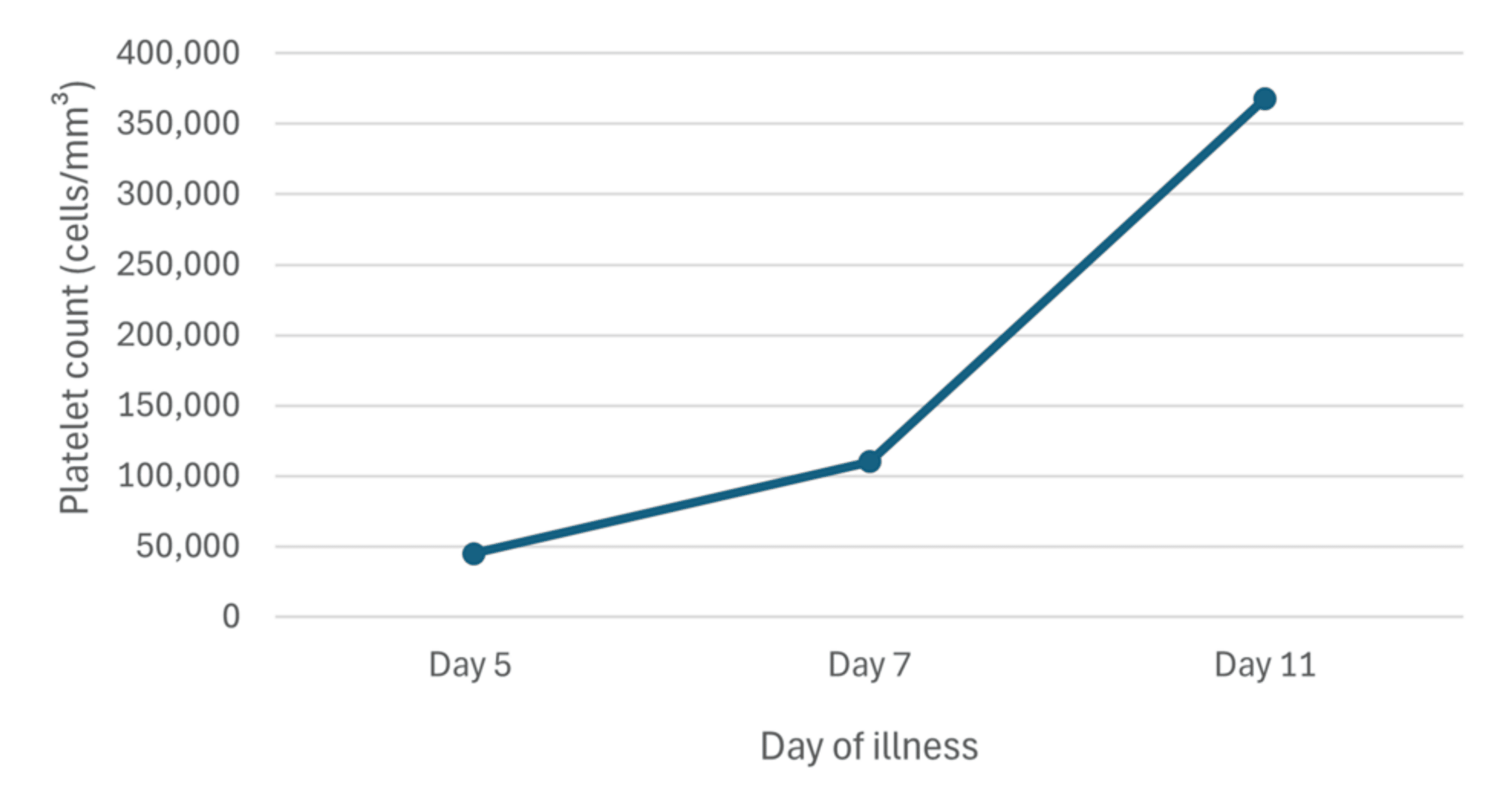
Evolution of the patient's platelet count On day 5 of illness, rupatadine 40 mg/day was started, with subsequent improvement in platelet count (reference range: 150,000-450,000 cells/mm³).

During hospitalization, the patient remained hemodynamically stable and afebrile. On the second day of hospitalization, the patient’s general condition had notably improved, the platelet count increased to 110,000 cells/mm^3^, and there were no signs of active bleeding. Due to the patient’s clinical improvement and in order to prevent hospital-acquired infection by SARS-CoV-2, the patient was discharged from the hospital with ambulatory treatment, completing five days of rupatadine treatment at home. Treatment with rupatadine was well tolerated, with no reported adverse effects. On the 11th day of illness, the patient's platelet count was 368,000 cells/mm^3 ^(Figure 1).

## Discussion

This case presents the effectiveness of the off-label use of oral rupatadine (40 mg/day) for two consecutive days in a hospital setting, in a real context of severe dengue infection. The rapid and sustained improvement of the patient’s clinical course with rupatadine is consistent with the results of a phase 2 randomized, double-blind, placebo-controlled trial carried out in outpatients with severe dengue in Sri Lanka [9]. In this study, 123 patients were treated with oral rupatadine 40 mg/day for five days, and 126 with a placebo, all of which were within ≤ 3 days since the onset of symptoms. Patients in the rupatadine arm were less likely to develop DHF than those in the placebo arm (9.7% vs. 19.5%) (relative risk 0.68, 95% CI 0.41-1.08), although the difference was not statistically significant (p = 0.09). However, rupatadine significantly reduced the percentage of patients with platelet counts < 50,000 cells/mm^3^ (p = 0.01), persistent vomiting and headache (p = 0.04), and hepatic tenderness (p < 0.0001), as well as the duration of illness (p = 0.0002). However, the trial had to be prematurely stopped, as the National Institute of Infectious Diseases (the trial center) had to be converted into a COVID-19 hospital in February 2020. It should be noted that in a post-hoc analysis of a preliminary randomized trial conducted by the same authors, treatment with rupatadine 40 mg/day was associated with higher platelet counts and lower AST levels on day 7 and smaller effusions on day 8 compared to the placebo group [9].

Our patient received 40 mg/day during the two-day hospitalization period, which was well tolerated and resulted in rapid improvement of symptoms and a dramatic recovery of thrombocytopenia. Besides the PAF inhibitory effect, rupatadine also blocks histamine receptors, inhibits degranulation of mast cells and the release of leukotrienes [10-13], and given that mediators released from mast cells contribute to vascular leak in dengue, these additional effects of rupatadine could possibly be involved in the efficacy of the drug in DHF [9]. Rupatadine has demonstrated, in vitro, its unique capacity to block the effects of mast cell secretions, and treatment with 20 mg of rupatadine was effective in patients with mast cell activation disorders [14].

Dengue infection is characterized by fever, severe headache, muscle and joint pain, abdominal pain, nausea, and vomiting. In this case, the patient presented with fever for more than two days and three more symptoms (headaches, abdominal pain, and nausea), meeting national criteria for suspecting dengue [8]. However, the first disease to be discarded at that time (July 2020) was COVID-19 because of the transmission risk and the probability of complications for the patient. The similarities in clinical presentation between dengue and SARS-CoV-2 infection during the COVID-19 pandemic pose a challenge to physicians in effectively diagnosing and treating dengue infection [15]. Repeated COVID-19 testing in our patient was negative.

DHF is characterized by high temperature, hepatosplenomegaly, hemorrhagic phenomena, hypovolemic shock, and cardiovascular disturbances. According to the WHO criteria, the diagnosis of DHF requires evidence of plasma leakage, which in our patient was supported by the presence of clinical ascites, together with thrombocytopenia and hemorrhagic manifestations. The patient showed transvaginal bleeding, which, in the context of El Salvador, was highly suggestive of dengue, with warning signs supporting the need of inpatient management [16]. Although abdominal pain was a persistent clinical manifestation in our patient, positive dengue IgM and IgG antibodies were a clue to the diagnosis. Recently, a case of severe dengue with hyperinflammatory state-associated acute pancreatitis has been reported [17]. In a review of 22 studies with 9,365 dengue patients, 16% presented with an acute abdomen, 7.7% of which were attributed to an acute pancreatitis [18]. Acute abdomen is an uncommon presenting form of dengue, and a high degree of suspicion of dengue fever is necessary when a patient from a dengue-endemic area presents with acute abdomen, including cholecystitis, acute appendicitis, appendicular perforation, splenic rupture, or nonspecific peritonitis [18]. The absence of imaging studies, due to healthcare restrictions during the COVID-19 pandemic, may be considered a limitation. However, the clinical findings were sufficient to support the diagnosis.

In cases of primary dengue infection, the IgM antibodies become detectable on days 3-5 of illness and persist for 2-3 months, whereas IgG antibodies appear by the 14th day and persist for life [19]. Secondary infection shows that IgG rises within 1-2 days after the onset of symptoms, simultaneously with IgM antibodies. The patient showed the presence of both IgM and IgG antibodies on the fifth day of the disease, indicating the probability of previous dengue infection. It is well described that a second infection with a different DENV serotype (heterotypic infection) can generate antibodies with a lack of capability of viral neutralization and could cross-react with heterotypic DENVs, triggering an antibody-dependent enhancement (ADE). This immune phenomenon is believed to be one of the major underlying mechanisms during immune-recall responses leading to increased severity in secondary DENV infection [20].

## Conclusions

The present case shows that the addition of rupatadine 40 mg/day for two days to conventional supportive fluid replacement and analgesic therapy was effective in a female adolescent from El Salvador diagnosed with DHF, who was likely experiencing a second infection. The rapid improvement in clinical symptoms and recovery from thrombocytopenia with rupatadine supports the involvement of PAF-related mechanisms in dengue infection and suggests a potential key role for potent PAF antagonists in the management of severe forms of the disease.
